# POWAINDv1.0: A Program for Protein-Water Interactions Determination

**DOI:** 10.6026/97320630014530

**Published:** 2018-12-22

**Authors:** Sahini Banerjee, Buddhadev Mondal, Rifat Nawaz Ul Islam, Parth Sarthi Sen Gupta, Debanjan Mitra, AmalKumar Bandyopadhyay

**Affiliations:** 1 Department of Biological Sciences, ISI, Kolkata, West Bengal, India; 2 Department of Zoology, Burdwan Raj Collage, East Burdwan, West Bengal, India; 3 Department of Zoology, The University of Burdwan, East Burdwan, West Bengal, India; 3 Department of Biotechnology, The University of Burdwan, East Burdwan, West Bengal, India; 3 Department of Chemistry, IISER Berhampur, Ganjam, Odisha, India

**Keywords:** Crystallographic Shell-water, program, water dynamics, bridge interactions, residue-specific interactions, atom specific interactions

## Abstract

Protein is the most exposed biomolecule in the aqueous environment of the cell. Its structure maintains a delicate balance between the rigidity and the flexibility that imparts binding specificity to its substrate/ligand, etc. Intramolecular interactions of polar and non-polar groups of amino acid residues and intermolecular weak interactions between these groups and shell-waters may contribute to the overall stability of the tertiary structure. However, the question as to what are the dynamics of interactions of shell-water with respect to weak forces and atom-groups of protein (AGP), requires systematic investigations. In this end, we have developed a procedure POWAINDv1.0 that analyzes interactions of crystallographic shell-waters (CSH) in residues and AGP specific manner. The shell-water and AGP specific bridge-interactions are also extracted. Further, the program analyzes favorable and unfavorable nature of each interaction based on the actual and 75% of the sum of van der Waals (vdW) radii of interacting atoms. The EXCEL-outputs are useful in understanding the profile for AGP-CSH interactions and contribution of each component in AGP. Taken together, the program provides intricate details on CSHprotein interactions and finds application in the structural Bioinformatics

## Background

Water is the solvent for structure and stability of biomolecules 1[1]. Spontaneous folding of the protein (via hydrophobic collapse) is directed by the characteristic ordering of water molecules around the side-chains of non-polar residues. The solubility of a folded protein is due to the intermolecular interactions between various kinds of polar-atom-groups (PAGs) of protein and water molecules. Due to dipolar nature of water, it participates in various kinds of non-covalent interactions with PAGs including hydrogen bond (HB), electrostatic (ELS), and van der Waals (vdW) interactions 1. Water can either be as donor or acceptor of HB and as a negative or positive partner for ELS interactions. Surface composition of the folded protein is largely made by PAGs, which are the preferred partners for interaction with bound waters. Internal waters are also present in the core and cavity of proteins, which make ordered or disordered interactions with protein's atom groups. These interacting waters are known as crystallographicshell- water (CSH) or bound-water 2. CSH could be found in the catalytic-site, ligand-binding site, and hydrophobic-patches. These bound-waters may contribute to the specificity of interaction for these sites 2. CSH in the interior of a protein is also crucial for structural stability that generally forms bridge interactions 2. These bridge interactions could be of two types, first, a molecule of water may form multiple interactions with PAGs and second, PAG may make multiple interactions with many water molecules. The fact that the interior of protein may have unsatisfied donor or acceptor or same-charged PAGs in close proximity, such unfavorable situations may largely be circumvented by interiorly bound waters.

X-ray crystallography, NMR and molecular dynamics simulation are the available methods that potentially detect the presence of shell-water in protein molecules 3. While X-ray crystallography is suitable for the detection of ordered bound-waters in protein structure, NMR is efficient to localize disorderly bound-waters 2. Although in X-ray methods, diffraction of water's oxygen could be differentiated from noise, its efficacy depends on several factors such as "i] crystallographic resolution, ii] R factor, iii] percentage of solvent in the crystal, iv] average B factor of the protein atoms, v] percentage of amino acid residues in loops, vi] average solventaccessible surface area of the amino acid residues, vii] grand average of hydropathy of the protein(s) in the asymmetric unit and viii] normalized number of heteroatom that is not water molecule. Furthermore, additional factor like the type of software package used for the development of the x-ray model also plays a secondary role in the accuracy of bound waters" 4.

To gain insight into the water-protein interactions, one needs to understand PAGs of each amino acid. For example, if we consider ASP, it has two polar atoms in the main-chain (i.e. O and N) that may mediate polar interaction with water via HB. alpha-, beta- and gamma-carbons can mediate van der Waals interactions with water molecules. There are two delta-oxygens that can mediate HB and ELS interactions with waters, as -COOH dissociates as -COO(-) (Section 1, sm) in the cellular aqueous medium. Overall, the neutral, positively and negatively charged PAGs of 11 amino acids (DESTRKHYNQC) forming the complete subset for polar interactions. In these aspects, the following questions are noteworthy. Which PAG/residue preferably binds more waters? Which PAG/residue dominates in HB/ELS interactions over others? Which PAG/residues involve in maximum bridge interactions? These questions are relevant in understanding the role of a polar component of protein in protein-water interactions in the surface and in the core of the protein. Similarly, the role of non-polar atom groups (NPAGs) is required to be understood. It is, however, difficult to address these interactions manually. At present, there are about 1.3 lacs of protein structures in the RCSB database of which 26,000 have small molecule ligands. Almost all of these structures possess CSH as an essential component of the structure. Since the input data is highly enriched in the database, and since the questions raised above are highly relevant, systematic extraction of PGAs and NPGAs specific water-protein interactions using appropriately screened (e.g. resolution and see above) dataset appears to be demanding.

In this work, we present details on our procedure, POWAINDv1.0 that function in CYGWIN-32bit UNIX like operating system in a window environment. It takes single crystal structure (may have multiple chains) and multiple distance-containing file as input to redirect outputs, which reveal detailed insight into the CSH-AGP interactions. While flow-chart shows operational details of the program, analysis of the results using a representative crystal structure provide application and scope of the program. Overall, the program provides details on CSH-AGP interactions and finds application in structural bioinformatics.

## Methodology

### The operation principle of the program

**Figure 1:**
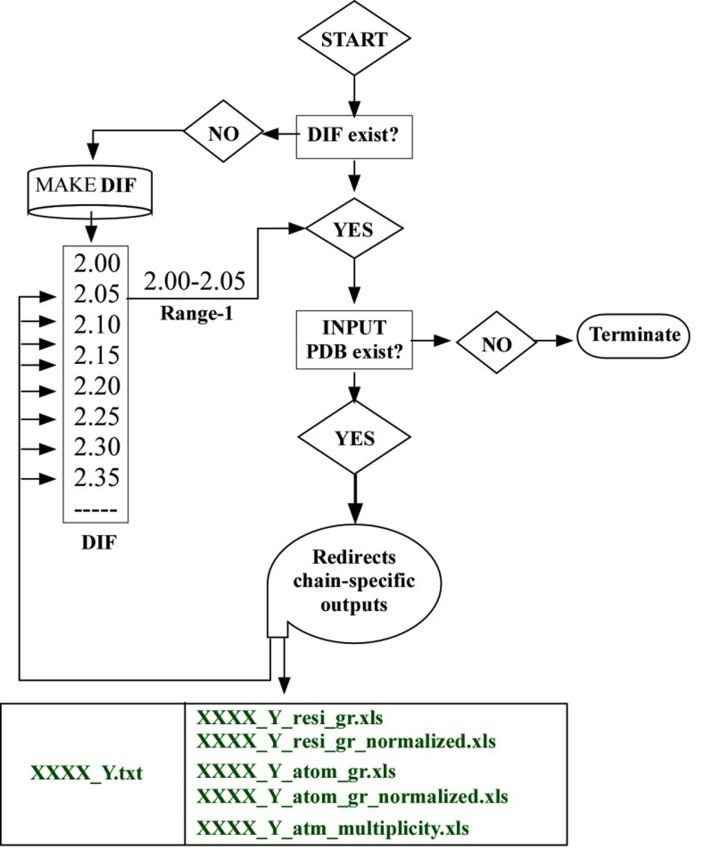
Flow chart of the functioning of the program POWAINDv1.0. DIF: Distance input file; XXXX: four-letter code of X-ray PDB file, where Y is the chain of the PDB file.

Upon start of the program, it verifies the directory structure and distance-input-file (DIF) in the working directory (Figure 1). In the first case, it terminates and in the second case, it produces a model DIF file, which user can edit and rerun the program. If the input PDB file is (i.e. XXXX.pdb) not found, the program terminates. The PDB file has only one chain i.e. Y in the present case. The program performs chain-specific and range-specific analysis of CSH-AGP interactions. Upon completion of one range (i.e. 2.00-2.05), it goes to the second (i.e. 2.05-2.10) and so on. Upon completion of all ranges for one chain, POWAINDv1.0 goes for next chain and so on, until all chains are exhausted (not shown). Three outputs in EXCEL format are redirected per chain. Residue-specific and atom-specific interactions are redirected as XXXX_Y_resi_gr.xls and XXXX_Y_atom_gr.xls files respectively. Normalized outputs for these files are also produced as XXXX_Y_resi_gr_normalized.xls and XXXX_Y_atom_gr_ normalized.xls. The normalization is done by using the formula: (interaction_frequency*100)/ (frequency_of_atom_group in the protein chain). The program also redirects chain-specific details on bridge-interactions in XXXX_Y_atm_multiplicity.xls file. Two types of bridge interactions are presented in the file. A text output is also produced, where range specific details are presented. It is useful to understand the favorable and unfavorable nature of interactions (see below).

## Run of the program:

The program runs in CYGWIN 32bit OS in a window environment from C/B-shell. Before the run of the program, the input distance file ("inp_dist") is to be edited as per the requirement. The program can interpret distances up to third decimal points, which may be required to increase the precision of analysis and dynamics of interactions of water as a function of weak forces (Section 2, sm).

## Program input:

Two inputs are necessary for a successful run of the program. First is a protein data-bank (PDB) file in X-ray format and the second is the input distances in the "inp_dist" file. Although, the program can analyze an X-ray structure file with any number of chains, such PDB files not necessarily provide details on water-protein interactions for the sites of protein-protein, protein-ligand interactions. Such a problem could be avoided by the choice of monomeric protein structure, apart from other precautions (see above).

## Program Output:

The output of POWAINDv1.0 includes three excel files and a distance-range-specific text file. Excel files include distance-rangespecific frequency of bound-water for i] residue-types and ii] atomtypes of protein. These two outputs are also repeated to give normalized outputs of these items. Third excel file details on distance-range-specific bridge-interactions. The text file is a distance-range-specific file that reports favorable/unfavorable interactions along with other details (Section 4, sm).

## Case study:

To understand the functioning, reliability, and interpretation of outputs of POWAINDv1.0, we performed detailed analysis on 3E9L.pdb, a monomer with resolution 1.95Å; UniProt ID Q6P2Q9. The protein, 3E9l is the domain of human Pre-mRNA-processingsplicing factor 8, for the range 1755-2016. It has 257 residues that bind 315 moles of crystal waters.

## General characteristics:

3E9l has been analyzed using PHYSICO2 5,6 and Cosurim 7,8 to understand the sequence and structure properties of the protein (Table 1). Following points are noteworthy. First, the aliphatic Index shows the protein is compositionally stable. The pI of the protein is at the neutral range and similar to the pH of cytoplasm. However, as the subcellular location of the protein is the nucleus, its solubility may not be an issue. The GRAVY is almost close to zero, indicated that there exists a balance between hydrophobic and hydrophilic composition in the protein. Second, residues in the core and surface of the protein are seen to be 46.8% and 53.2% respectively. Although the majority of core residues are hydrophobic, it also has hydrophilic residues. Similar is the case for the surface (Table 1). Such mixed-type-composition in the core and surface may have differential effects in interactions with shellwaters (see below). Third, Analysis of sequence BLOCK using APBEST 9 shows that the NCS: CS (non-conservative to conservative) is 0.32 indicated that the divergence of the protein is low. The fact that RNA binding proteins are more conserve 10 and 3E9l (Q6P2Q9) is a nuclear RNA binding protein that mediates spliceosome formation; the low NCS:CS is not surprising. Both candidate-residues of the dominant herero-pair are hydrophobic (Table 1). This suggests that the functional constraint seems to be more contributed by hydrophobic but not hydrophilic residues.

Forth, since we were interested to explore the interactions between shell-water and protein, and since salt-bridge/ion-pair 11,12 are also involves in these interaction process, we have investigated ionpair interactions 11,12 and their energetics using automated procedure 13,14 involving APBS 15 and PDB2PQR 16. These analyses show that salt-bridges (SB) are very few in the protein. The binary analysis of networked vs isolated, core vs surface, nonlocal vs local, HB vs non-HB and Stabilizing vs destabilizing saltbridge shows that the former items have a greater contribution than the latter. Taken together, these analyzes suggest that 3E9l is a conserve and stable protein with a balanced distribution of polar and non-polar residues in the core and surface. How crystal waters make interactions with these polar and non-polar residues? Do saltbridge forming residues interact with shell-water?

## Distance-dependent profiles of CSH-AGP interactions:

**Figure 2:**
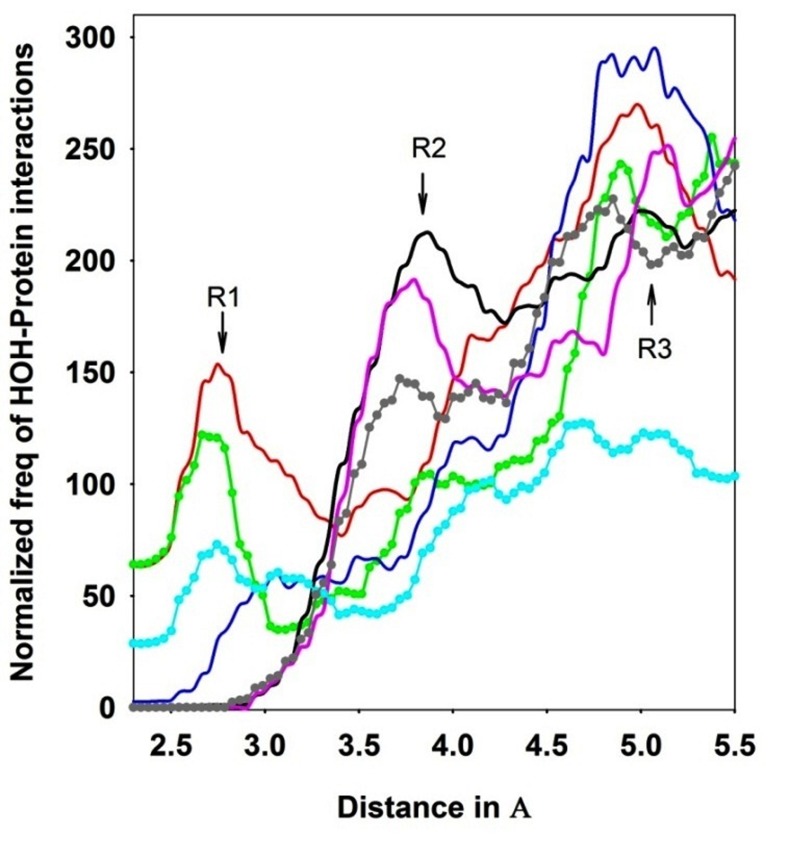
Normalized protein-water (HOH) interaction profiles for polar-side chains (red), oxygen-atom (green) of main-chain, nitrogenatom (blue) of main-chain, side-chain of acidic (D, E) and basic (R, K) residues (cyan), carbon-atom of main-chain (pink), non-polar aromatic (grey) and non-polar all atoms (black) of all residues. The auto-generated normalization output that uses the formula, (actualinteraction- freq*100)/(freq-of-atom-group in protein chain) was used. The polar-atoms of ASP (OD1, OD2), GLU (OE1, OE2), ASN (OD1, ND2), GLN (OE1, NE2), SER (OG), THR (OG1), TYR (OH), LYS (NZ), ARG (NH1, NH2, NE), HIS (ND1, NE2), THP (NE1), MET (SD) and CYS (SG) (i.e. total 20 atoms-types) are considered. Main-chain oxygen (20 O-atoms for 20 residues) and nitrogen (20 N-atoms for 20 residues) are separately considered. For the plot of non-polar atom-types, C (of main-chain), C alpha (of main-chain), C beta, C gamma, C delta, C epsilon, C zeta, C eta for all residues (total 107 atom-types are present in protein-chain) are considered. As the number of atom-types in non-polar groups is 107 types, to scale the profile of non-polar atom-types as other-types (such as polar, main-chain oxygen, etc that have about 20 atom-types), it is divided by 5. Non-polar main-chain-carbon (total 20 for 20 residues) profile is separately plotted (Section 4, sm).

To address the above-mentioned issues, we have analyzed 3E9L.pdb using POWAINDv1.0. The profile of normalized HOHprotein interactions by various atom-types (side-chain polar, mainchain oxygen, main-chain-nitrogen, main-chain-carbon, and nonpolar carbon and other types) are presented in Figure 2. Several observations are noteworthy from the figure. 

**Figure 3:**
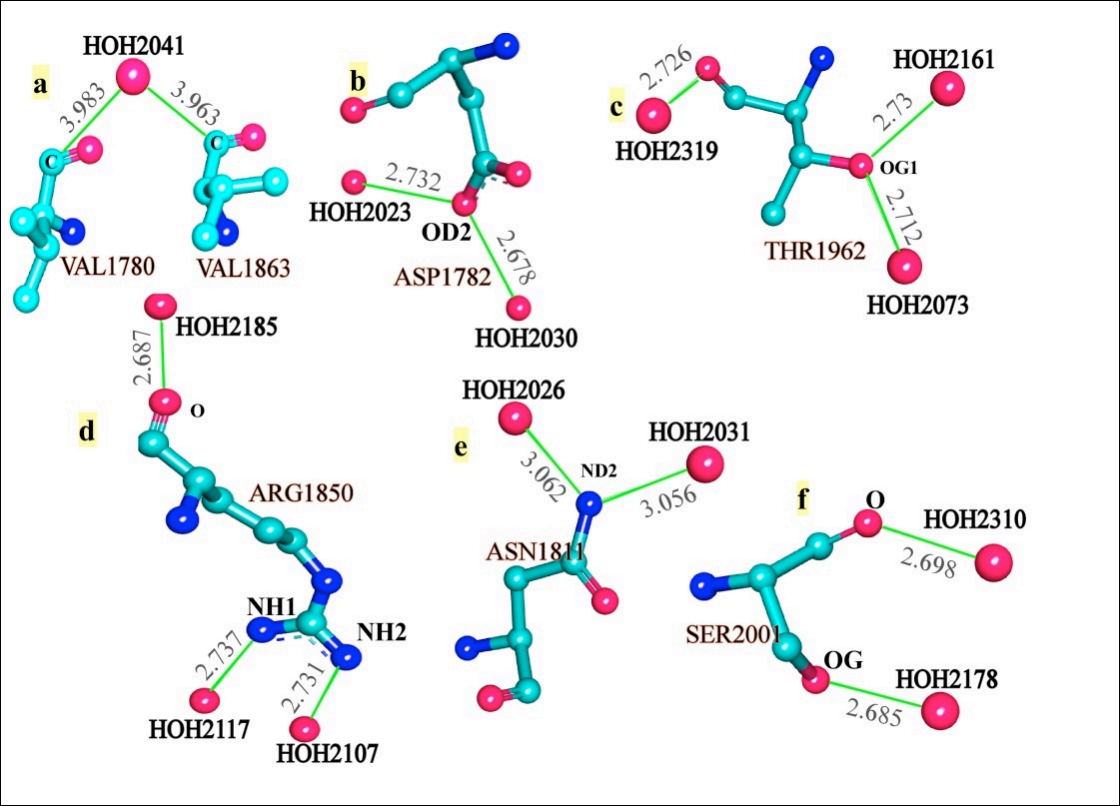
Representative atom-types and their interaction with crystal waters. Distance, atom-type ID and other details are directly extracted from the program-generated output.

First, the profile for polar atom-types (Figure 2, red) broadly shows two optima, one at R1 and other at R3 region. The first region (R1) may be crucial for inter-dipole interactions (hydrogen-bond, electrostatic etc) between water and side-chain polar atom-types of protein (Figure 3, from b to f). Here, OD1, OD2 (ASP; Figure 3b), OE1, OE2 (GLU), OD1, ND2 (ASN; Figure 3), OE1, NE2 (GLN), OG (SER; Figure 3), OG1 (THR; Figure 3), OH (TYR), NZ (LYS), NH1, NH2, NE (ARG; Figure 3), ND1, NE2 (HIS), NE1 (TRP), SD (MET) and SG (CYS) are involved in the interaction with water molecules. Unlike the profile of main-chain nitrogen (N-type; blue profile), in R1, the main-chain oxygen (O-type; green profile) makes much greater contributions in the interactions.

The charged atom-types of acidic and basic (cyan) also contribute. It is interesting to note that the non-polar atom-types (C-type; main-chain only C, in pink, aromatic non-polar types in grey and all non-polar types in black) have no interactions for the region. Second, the profile for the C-type (Figure 2, pink), non-polar aromatic (Figure 2, grey) and all non-polar (Fig. 2, black) atomtypes are showing a maxima ~3.9Å, which is absent for polar atomtypes, O-type, charged-side-chain of acidic and basic atom-types (cyan) and N-type. This region seems to be dominated by C=O/CH mediated hydrogen bonding 17 and p-system of C=C mediated interactions with water 18. Notably, hydrogen bond in this region (R2) seems to be weaker than the region R1. The fact that both the profiles of all non-polar atom-types (total 107 types) and C-type (total 20, for 20 residues) have been similar and are almost of equal amplitude (after dividing the former by 5), it could be said that the majority of these interactions are similar type (Figure 3a). The Ctype, which is attached to oxygen, may be forming a hydrogen bond with water atoms. These interactions seem to be useful for the stability of the protein.

Third, the region R3 is centered on 5Å, which is beyond hydrogen bonding limit. The region is suitable for ion-pair, van der Waals, cation-p and anion-p types of interactions 19. PHE, TYR, and TRP may interact with water dipole via their p-ring systems. It is noteworthy; that the aromatic atom-types profile (Figure 2; grey) is almost similar as black and pink profiles, indicated that the former makes major contributions to the non-polar groups mediated water-protein interactions. The -NHCO- group of main-chain may play important role in polar interactions with water dipole as the maxima for both N-type and O-type is at the highest level in this region. While ion-pair interactions between the water dipole and side-chain of basic (positively charged) and acidic (negatively charged) atom types seems to be the contributor (Fig. 2, red) in R3 region, other types of interactions may be involved as the former profile (Figure 2, cyan), is much lower than the resultant profile (Figure 2, red) in this region (Section 4, sm).

## Differential contributions of components of AGPs of a specific type

**Figure 4:**
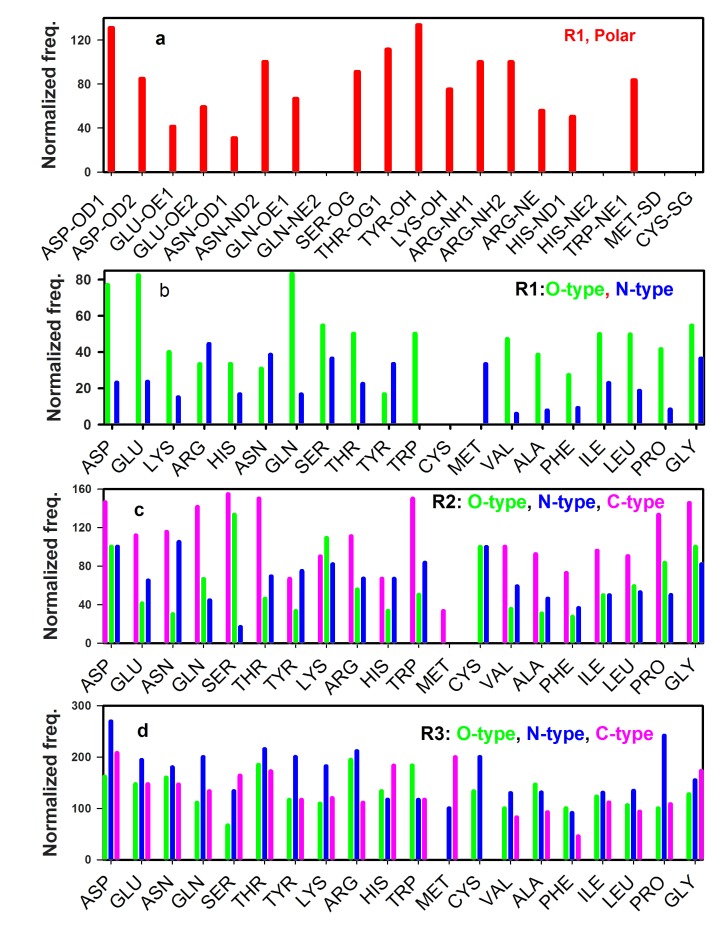
Plot of components of different types for different regions.

Binding of waters by different amino acid residues depends on many factors such as their side-chains, location in proteins structure 20. Each distance specific region (R1, R2, and R3) contributed by different atom-types (such as polar, O-type, N-type etc). How components in each type contribute in the netinteraction? To address this question, we have plotted region specific few types with details of their components in Fig. 4. Several points are noteworthy from Figure 4.

First, of all components of polar type (Figure 4), OD1 of ASP and OH of TYR are the highest contributors. Both NH1 and NH2 of ARG, make equal contributions. NE2 of GLN, NE2 of HIS, SD of MET and SG of CYS has no contribution at all (Figure 4) in the protein studied here. Second, in region R1, O-type (Figure 4b, green) has a greater contribution for most of the residues than Ntype (Figure 4, blue). Third, in region R2, C-type (pink) has a greater contribution (Figure 4) than O-type (green) and N-type (blue). This is also reflected in the respective profiles in Figure 2, where Ctype (pink profile) dominates over O and N-types. Forth, in R3, the order is seen to be N-type>O-type>C-type. These region-specific variations indicate an overall change of interactions between water and different atom-types of protein (Section 4, sm).

## Bridge-interactions between CSH and AGP

**Figure 5:**
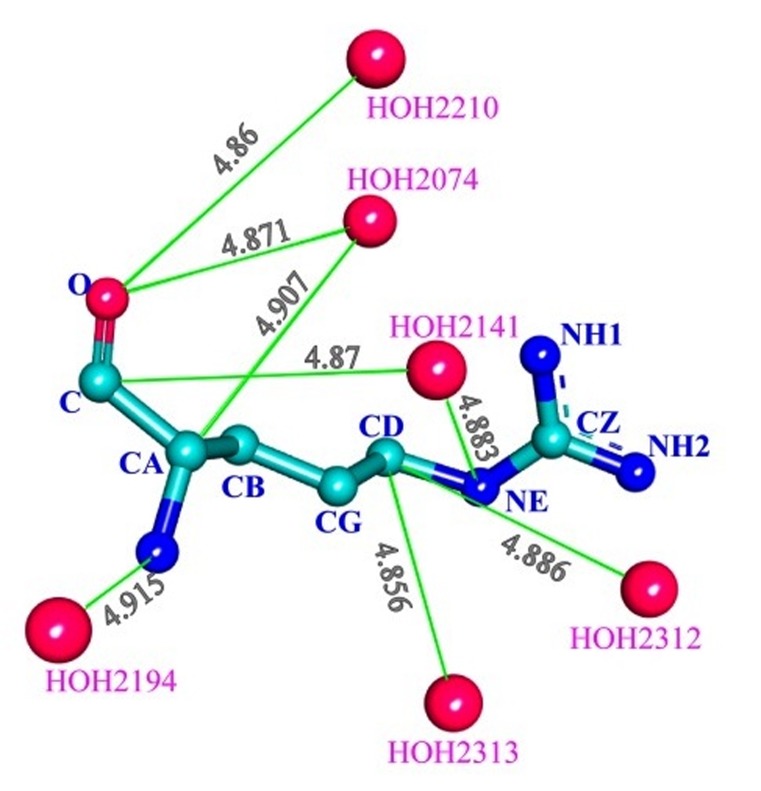
Bridge interactions of ARG1949 with different water molecules

In water-protein interactions, the most interesting observations are the bridge interactions, which are known to contribute to the overall stability of local structure and topology 2,21. The program extracts all possible details of such bridge interactions. Two types of bridge interactions are seen to occur in this case. First, a water molecule is making interaction with various atom-groups of the same/different amino acid residues of protein (Figure 3). Second, an amino acid residue is making interactions with the different molecule of waters (Figure 3). Such bridge-interactions are increased from bivalent to multivalent with an increase in distance of interaction. NACCESS 22 [22] analysis shows that mainchain of the residue ARG1949 (Figure 5) is deeply buried and the side-chain is exposed to the surface of the protein. It is seen that both the main-chain and the side-chain bind multiple water molecules (Section 4, sm).

## Conclusion

We have developed a program POWAINDv1.0 that takes a PDB file and a distance file as input and redirects four types of outputs. Residue-specific and atom-type-specific absolute and normalized frequency of water-protein interactions is written in a range-ofdistance specific manner. Different types of bridge interactions are separately redirected in EXCEL file. A text output is also produced with details on residues of a protein in the range of distance specific manner. The case study with 3E9L.pdb using the program shows a number of interesting observations. While polar and nonpolar interactions are dominated in short (≤3.5Å) and intermediate ranges (≤4.3Å) respectively, both these interactions making almost equal contributions at long range (≤5.5Å). Further insight is revealed in the analyses of the contribution of components in each of this type (e.g. polar-type, non-polar type, N-type etc). The range of distance specific analysis of bridge interactions would highlight the importance of crystal water. Taken together, the program provides details on protein-water interactions, the knowledge of which seems to have potential applications in protein engineering and structural bioinformatics.

## Figures and Tables

**Table 1:** Sequence and structural properties of 3E9L.pdb (UNIPROT ID Q6P2Q9)

Sequence analysis; ID Q6P2Q9; length 257 residues																						
Aliphatic Index														112.26								
Iso-electric point (pI)														7.06								
Grand Average Hydropathy (GRAVY)														-0.06								
Evolutionary conservation analysis; BLOCK (Length=54; Width=256)																						
Non-conservative (NCS) : Conservative (CS) substitution														0.32								
Most dominant hetero-pair										ILE-VAL (IV)												
Core and Surface distribution of residues																						
Str	Hydrophobic* (VAFILMCPG)									Hydrophilic* (STNQDERKHWY)												
Residue	V	I	L	M	C	F	A	P	G	D	E	R	K		H	N	Q	S	T	W	Y	T
Abs (co)	7	20	26	2	1	10	9	5	5	5	2	3	3		2	3	2	4	3	5	3	120
% (co)	2.7	7.8	10.1	0.8	0.4	3.9	3.5	1.9	1.9	1.9	0.8	1.2	1.2		0.8	1.2	0.8	1.6	1.2	1.9	1.2	46.8
Abs (su)	10	6	6	1	0	1	4	7	6	8	15	6	17		4	10	10	7	15	1	3	137
% (su)	3.9	2.3	2.3	0.4	0	0.4	1.6	2.7	2.3	3.1	5.8	2.3	6.6		1.6	3.9	3.9	2.7	5.8	0.4	1.2	53.2
Salt-bridge (SB) and SB-energy analysis																						
Networked vs Isolated										5 vs 4 i.e. Total=9												
Core vs surface										5 vs 4 i.e. Total=9												
non-local vs Local										6 vs 3 i.e. Total=9												
HB SB vs non-HB										5 vs 4 i.e. Total=9												
Stabilizing vs destabilizing										7 vs 2 i.e. Total=9
